# Moderating effects of psychological factors and frequency of experiences in the emergency department: The role of perceived quality of healthcare

**DOI:** 10.1097/MD.0000000000035134

**Published:** 2023-09-15

**Authors:** Alina Abidova, Pedro Alcântara da Silva, Sérgio Moreira

**Affiliations:** a National School of Public Health, NOVA University of Lisbon, Lisbon, Portugal; b Institute of Social Sciences, University of Lisbon, Lisbon, Portugal; c Faculty of Psychology, University of Lisbon, Lisbon, Portugal.

**Keywords:** emergency department, frequent users, level of happiness, level of satisfaction with life, perceived quality of healthcare, psychological factors

## Abstract

The aim of this study is to identify the main moderators in the relationship between antecedents/predictors (doctors, privacy, accessibility, and availability, perceived waiting time to be called back by the doctor after the examinations and/or tests) and the perceived quality of healthcare (PQHC) in the emergency department (ED). Patients admitted to the ED of a public hospital in Lisbon, Portugal, between January and December 2016 were included in this study, with a representative sample size of 382 patients. A 5% margin of error and a 95% confidence interval were used, and all data were collected between May and November 2017. We used a stepwise multiple linear regression analysis to test the moderation models. We identified 3 main moderators with different moderating roles between the antecedents (predictors) and PQHC: level of life satisfaction, level of happiness, and frequency of ED experiences. Overall satisfaction with doctors is more likely to influence the PQHC among patients with lower levels of life satisfaction. Moreover, privacy and perceived waiting time to be called back by the doctor after an examination and/or test are more likely to influence the PQHC among patients with lower levels of life satisfaction and happiness. Finally, accessibility and availability are more likely to influence the PQHC among patients with more frequent ED experiences. Thus, knowing the moderating effects of psychological factors and the frequency of ED experiences may help to better understand the relationship between PQHC and certain predictors.

## 1. Introduction

Perceived service quality can reflect a patient’s impression regarding the overall service provided by healthcare providers.^[1]^ Care quality can be assessed by the extent of improvement in a patient’s physiological functions as a result of receiving medical care services.^[2]^ To evaluate the quality of a service, patients consider many different aspects of medical care.^[3]^ Among these, research has highlighted the importance of sociopsychological factors in quality assessments.^[1]^

Psychological well-being, which includes subjectively measurable aspects, such as morale, happiness, and life satisfaction,^[4]^ is associated with health-related behavior and frequent attendance.^[5]^

Life satisfaction describes a person’s cognitive assessment of their overall satisfaction with various aspects of life,^[6]^ and it can serve as a potential predictor of morbidity and mortality.^[7]^ Various studies have associated lower mortality rates and fewer chronic health conditions with higher levels of life satisfaction.^[8,9]^ At the same time, higher healthcare utilization has been associated with higher levels of life dissatisfaction.^[10]^

Happiness is defined as a state wherein positive feelings or emotions are experienced more intensely and frequently than negative ones. Happiness serves as a protective mechanism against psychological disorders and helps achieve positive outcomes. In this sense, happy individuals perceive things differently.^[11]^

Generally, a high level of subjective well-being is associated with positive outcomes in many life areas.^[12]^ Happy individuals are more successful than less happy ones in 3 main life domains: relationships, work, and health.^[12]^ Happiness is considered to be vital for maintaining health.^[12]^ There is also a positive association between life satisfaction and health, suggesting that better health is associated with higher levels of well-being and vice versa.^[7,13]^

High levels of general life satisfaction create a positive outlook that can lead to patient satisfaction with care.^[14]^ Several studies have shown that life satisfaction is an accurate and sufficient concept that can be used to determine a patient’s quality of life and assess the quality of health services.^[15,16]^

Thus, it is important to more deeply understand what moderates patient perceptions of healthcare quality in the emergency department (ED). In this study, we seek to determine the effect of moderators (level of life satisfaction, level of happiness, and frequency of ED experiences) on the relationship between predictors (antecedents) and the perceived quality of healthcare (PQHC).

## 2. Methods

To calculate our random probabilistic sample size, we used a list of 55,903 patients who were admitted to the ED of a public hospital in Lisbon, Portugal, at least once between January 1 and December 31, 2016. All responders were at least 18 years old, able to answer the questions, residents of Portugal, and Portuguese-speaking. We excluded respondents under 18 years old who were unable to answer the questions, who resided outside Portugal, or who had psychiatric illnesses.

In cases where the individual chosen was admitted more than once to the ED in the year under study, we chose the last admission according to the date of admission. A 5% margin of error and a 95% confidence interval were used. The representative sample size comprised 382 patients, and the data were collected between May and November 2017.

A sample distribution by age and gender was calculated using several steps. First, we calculated the distribution of the universe with a total number of 55,903 patients. Second, we calculated an ideal distribution from the random, probabilistic sample selection of 382 individuals. Our gender distribution was ultimately sufficiently close to an ideal distribution, with a female prevalence.

Our age distribution was harder to control, and here we observed a prevalence of the 31 to 40 group of patients in our case and the 18 to 30 group of patients in the case of ideal distribution, while the 41 to 50 age group and the 80+ age group were sufficiently close to an ideal distribution.

Before sending the questionnaire, we contacted all patients by telephone to obtain their permission to send the questionnaire and consent to participate in the survey. We made telephone calls 3 times during the day at different times of the day. If our attempts to reach a patient were unsuccessful, the patient was classified as not responsive. During the data collection period, we made a total of 4,413 telephone calls, just including the first-call attempts and excluding all repeat calls afterwards. Those who did not have a telephone number on our list were excluded prior to the initiation of the calls. The questionnaire was sent by either mail or e-mail, depending on the respondent’s preference.

To carry out the analysis, we selected only the main predictors of PQHC that we considered to exhibit statistically significant conditions (*P* ≤ 0.05), as well as some other predictors that had a statistically significant (marginal effects) relationship with PQHC (*P* ≤ 0.10). We computed the moderation models for the PQHC considering the fact that both statistically significant predictors and moderators are significantly correlated with the dependent variable.

Notably, all the variables measuring more than 1 item were simplified into a single composite measure by using an exploratory factor analysis: (1) accessibility and availability (including the location of the hospital and ED within the city, the orientation within the ED, the distance between the different areas of the ED, and the availability of equipment and specialist staff to conduct tests, blood tests) and (2) doctors (including the doctor’s friendliness and helpfulness, the doctor’s competence and professionalism, how the doctor explained a health problem (diagnosis) during the examination, the explanations provided by the doctor on the exams performed and the objectives of the treatment to be undertaken, the information provided by the doctor on the precautions to be taken, and the doctor’s recommendations and instructions on how to take or apply the medications prescribed, written or oral, after leaving the hospital).

We performed an exploratory factor analysis using the principal axis factoring method for extraction, the scree plot for selecting the number of factors, and the Oblimin rotation for interpreting the factor loadings. Internal consistency analysis revealed Cronbach alpha values of 0.87 (accessibility and availability) with 54.5% of explained variance and of 0.98 (doctors) with 88.8% of explained variance. Thus, high alpha coefficients emphasize the conclusion that the items have good internal consistency, which in turn confirms that our measures are reliable and correct. Stepwise multiple linear regression analysis was used to test the moderation models along with the methodology proposed by Baron and Kenny (1986).^[17]^

## 3. Results

The participants were mostly from Lisbon (96.0%), comprising 61.3% females and 38.7% males. The age distribution of the participants across age groups was almost uniform: 18 to 30 years (14.9%), 31 to 40 years (19.1%), 41 to 50 years (14.4%), 51 to 60 years (17.6%), 61 to 70 years (9.2%), 71 to 80 years (9.8%), and 80+ years (14.7%). Table 1 shows the descriptive statistics of the main variables included in the moderation models.

**Table 1:** Descriptive statistics (mean, minimum, maximum, and standard deviation).

	n		Mean	Min	Max	SD
**Accessibility and availability**						
Location of the hospital and ED within the city	379		8.20	1	10	1.96
Orientation within the ED	374		7.44	1	10	2.05
Distance between the different areas of the ED	363		7.46	1	10	1.92
Availability of equipment and specialist staff to conduct tests, blood tests	366		7.32	1	10	2.19
Overall accessibility and availability	375		7.49	1	10	2.08
**Privacy**						
How privacy was safeguarded	372		7.27	1	10	2.41
**Doctors**						
Friendliness and helpfulness of the doctor(s)	379		7.74	1	10	2.17
Competence and professionalism of the doctor(s)	374		7.90	1	10	2.15
How the doctor(s) explained a health problem (diagnosis) during the examination	378		7.78	1	10	2.30
The explanations provided by the doctor(s) on the exams performed and the objectives of the treatment to be undertaken	366		7.77	1	10	2.39
The information provided by the doctor(s) on precautions to be taken, as well as their recommendations and instructions on how to take or apply the medications prescribed, written or oral, after leaving the hospital	370		7.95	1	10	2.23
Overall performance of the doctor(s)	378		7.89	1	10	2.26
**Waiting time to be called back by the doctor (perception**)						
Waiting time to be called back by the doctor after the examinations and/or tests in view of the severity of the condition	314		5.58	1	10	2.71
**PQHC**						
Overall evaluation of the quality of healthcare	373		7.65	1	10	2.10
**Life satisfaction**						
Level of life satisfaction (in general)	375		7.58	1	10	1.97
**Happiness**						
Level of happiness (considering all aspects of life)	369		7.58	1	10	1.96
	**n**	**%**	**Mean**	**Min**	**Max**	**SD**
**Frequency of ED experiences**						
Number of times in the ED in 2016			2.21	1	20	2.22
1	121	47.1				
2	75	29.2				
3	24	9.3				
4	16	6.2				
5 or more	21	8.3				
Total	257	100				

**Figure 1.**
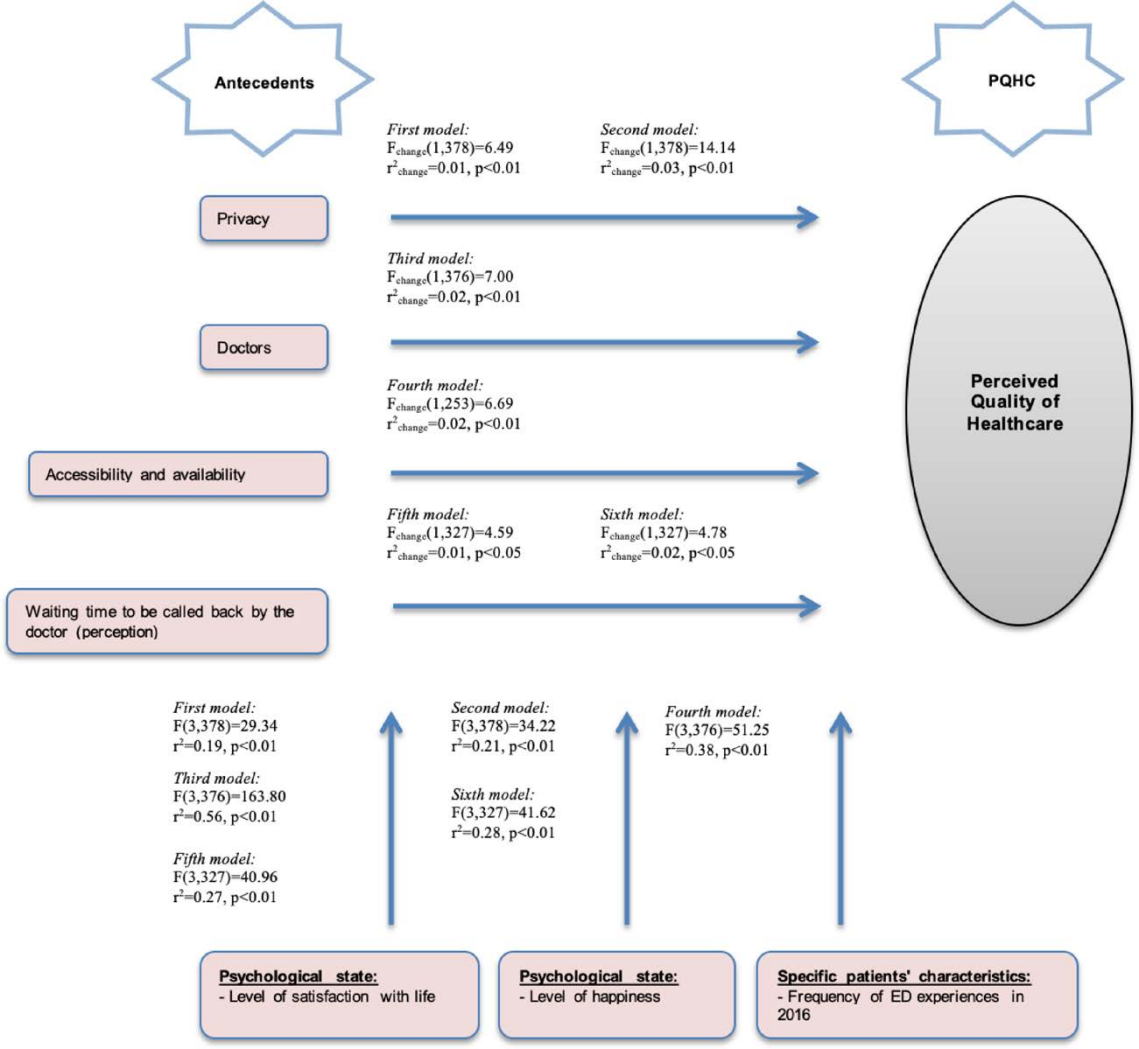
Moderation models for the perceived quality of healthcare.

The models’ descriptions and the statistical data are presented in Figure 1 and Table 2 format for better understanding and observation. As demonstrated in Figure 1 and Table 2, we considered only the moderation models that were statistically significant.

**Table 2:** Moderation models for the perceived quality of healthcare.

Model	Predictor	Moderator	Outcome	Moderation effect	Interaction effect (full model)
**1**	Privacy	Level of satisfaction with life	PQHC	F_change_(1,378) = 6.49 r^2^_change_ = 0.01, *P* < .01	F(3,378) = 29.34 r^2^ = 0.19, *P* < .01
**2**	Privacy	Level of happiness	PQHC	F_change_(1,378) = 14.14 r^2^_change_ = 0.03, *P* < .01	F(3,378) = 34.22 r^2^ = 0.21, *P* < .01
**3**	Doctors	Level of satisfaction with life	PQHC	F_change_(1,376) = 7.00 r^2^_change_ = 0.02, *P* < .01	F(3,376) = 163.80 r^2^ = 0.56, *P* < .01
**4**	Accessibility and availability	Frequency of ED experiences in 2016	PQHC	F_change_(1,253) = 6.69 r^2^_change_ = 0.02, *P* < .01	F(3,376) = 51.25 r^2^ = 0.38, *P* < .01
**5**	Waiting time to be called back by the doctor (perception)	Level of satisfaction with life	PQHC	F_change_(1,327) = 4.59 r^2^_change_ = 0.01, *P* < .05	F(3,327) = 40.96 r^2^ = 0.27, *P* < .01
**6**	Waiting time to be called back by the doctor (perception)	Level of happiness	PQHC	F_change_(1,327) = 4.78 r^2^_change_ = 0.02, *P* < .05	F(3,327) = 41.62 r^2^ = 0.28, *P* < .01

The first and second models include privacy as a predictor and the level of life satisfaction and happiness as moderators. Overall, patients’ levels of life satisfaction and happiness contribute to 1.0% and 3.0% of the explained variance in the given models, respectively. Thus, the effect of privacy on PQHC is moderated by the level of life satisfaction and the level of happiness by 1.0% and 3.0%, respectively, which are statistically significant (*P* < .01).

Without the level of life satisfaction and the level of happiness as moderators, the effect of privacy on PQHC is explained by 18.0%. Different correlation levels can be observed for the interaction of privacy with PQHC (r_x_ = 0.39, *P* < .01 and r_x_ = 0.38, *P* < .01); level of life satisfaction and level of happiness with PQHC (r_m_ = 0.09, *P* < .06 and r_m_ = 0.17, *P* < .01); and privacy, level of life satisfaction, and level of happiness with PQHC (r_x*m_ = 0.12, *P* < .01 and r_x*m_ = -0.17, *P* < .01), thus revealing a statistically significant moderation effect. By analyzing the entire models, including this effect of interactions, we found that 19.0% and 21.0% of the explained variance are statistically significant (*P* < .01).

Simple slopes analysis showed that the effect of privacy on PQHC is higher among patients with lower levels of life satisfaction (b = 0.47) and lower levels of happiness (b = 0.49) than among those with higher levels of life satisfaction (b = 0.22) and higher levels of happiness (b = 0.17).

The third model includes overall satisfaction with doctors as a predictor and the level of life satisfaction as a moderator. In this model, level of life satisfaction contributes to 2.0% of the explained variance. Thus, the effect of overall satisfaction with doctors on PQHC is moderated by the level of life satisfaction by 2.0%, which is statistically significant (*P* < .01).

Without the level of life satisfaction as a moderator, the effect of overall satisfaction with doctors on PQHC is explained by 54.0%. Different correlation levels can be observed for the interaction of overall satisfaction with doctors with PQHC (r_x_ = 0.72, *P* < .01); level of life satisfaction with PQHC (r_m_ = 0.07, *P* < .05); and overall satisfaction with doctors and level of life satisfaction with PQHC (r_x*m_ = 0.09, *P* < .01), thus revealing a statistically significant moderation effect. By analyzing the entire model, including this effect of interactions, we found that 56.0% of the explained variance is statistically significant (*P* < .01).

Simple slopes analysis showed that the effect of overall satisfaction with doctors on PQHC is higher among patients with lower levels of life satisfaction (b = 0.83) than among those with higher levels of life satisfaction (b = 0.64).

The fourth model includes accessibility and availability as a predictor and the frequency of ED experiences as a moderator. In this model, the frequency of ED experiences contributes to 2.0% of the explained variance. Thus, the effect of accessibility and availability on PQHC is moderated by the frequency of ED experiences by 2.0%, which is statistically significant (*P* < .01).

Without the frequency of ED experiences as a moderator, the effect of accessibility and availability on PQHC is explained by 36.0%. Different correlation levels can be observed for the interaction of accessibility and availability with PQHC (r_x_ = 0.58, *P* < .01); frequency of ED experiences with PQHC (r_m_ = –0.12, *P* < .05); and accessibility and availability and frequency of ED experiences with PQHC (r_x*m_ = 0.13, *P* < .01), thus revealing a statistically significant moderation effect. By analyzing the entire model, including this effect of interactions, we found that 38.0% of the explained variance is statistically significant (*P* < .01).

Simple slopes analysis clearly showed that the effect of accessibility and availability on PQHC is higher among patients with more frequent ED experiences (b = 0.87) than among those with less frequent ED experiences (b = 0.64).

The fifth and sixth models include perceived waiting time to be called back by the doctor after the examinations and/or tests as a predictor and the level of life satisfaction and happiness as moderators. In these models, the levels of life satisfaction and happiness contribute to 1.0% and 2.0% of the explained variance, respectively. Thus, the effect of perceived waiting time to be called back by the doctor on PQHC is moderated by the level of life satisfaction and the level of happiness by 1.0% and 2.0%, respectively, which are statistically significant (*P* < .05).

Without the level of life satisfaction and the level of happiness as moderators, the effect of perceived waiting time to be called back by the doctor on PQHC is explained by 26.0%. Different correlation levels can be observed for the interaction of perceived waiting time to be called back by the doctor with PQHC (r_x_ = 0.49, *P* < .01 and r_x_ = 0.48, *P* < .01); level of life satisfaction and level of happiness with PQHC (r_m_ = 0.13, *P* < .05 and r_m_ = 0.13, *P* < .01); and perceived waiting time to be called back by the doctor, level of life satisfaction, and level of happiness with PQHC (r_x*m_ = -0.10, *P* < .05 and r_x*m_ = -0.10, *P* < .05), thus revealing a statistically significant moderation effect. By analyzing the entire models, including this effect of interactions, we found that 27.0% and 28.0% of the explained variance are statistically significant (*P* < .01).

Simple slopes analysis showed that the effect of perceived waiting time to be called back by the doctor after the examinations and/or tests on PQHC is higher among patients with lower levels of life satisfaction (b = 0.47) and lower levels of happiness (b = 0.47) than among those with higher levels of life satisfaction (b = 0.26) and higher levels of happiness (b = 0.26).

## 4. Discussion

Healthcare mainly focuses on meeting the social, physical, and psychological needs of patients.^[18]^ Many researchers have highlighted that the psychological well-being of the population as a whole is associated with health (self-rated health), socioeconomic factors, and social relationships.^[19]^ They identified social determinants and sociodemographic factors, such as educational level, marital status, sex, and age, as well as illness-related variables, such as comorbidities and triage category, as risk factors for psychological distress in patients admitted to the ED.^[20]^ Other studies have highlighted that psychological distress is closely linked to self-reported health.^[20,21]^ Positive experiences in the context of negative or traumatic events affect patients’ physical and psychological well-being.^[22]^ One of the mechanisms with which one’s psychological state directly affects their physical health is the effect of such a state on the immune system.^[22]^

Research on immune activity and induced mood supports a causal relationship between immune function (changes in the immune function) and positive mood.^[22]^ For example, a happy mood can promote disease-fighting mechanisms and health by boosting one’s level of optimism.^[22]^ Furthermore, people with a positive mood are less likely to ignore negative information that is self-relevant and important.^[23]^ In one study, a positive mood was found to be associated with fewer work absences, fewer calls to the doctor, fewer emergency room and hospital visits, and less medication use.^[24]^ Patients with a positive mood were found to be less likely to report pain 2 days later and on the same day.^[24]^ Furthermore, a positive mood on a particular day has been found to predict fewer hospital and emergency room visits on the next day.^[24]^

Frequent ED users were also found to be more likely to report a poor or fair health status compared to other ED users.^[25]^ More specifically, our results show that accessibility and availability are more likely to influence the PQHC among patients with more frequent ED experiences than among those with less frequent ED experiences.

Happy people are more likely to remember positive events than negative ones.^[26]^ However, successful outcomes do not merely follow from happiness or correlate with happiness. Rather, they are caused by happiness.^[22]^ In this sense, happy people with higher levels of life satisfaction tend to demonstrate fewer unpleasant physical symptoms and better self-reported health.^[27,28]^ This suggests that a person’s level of happiness, along with their social and economic status, can influence their perceived service quality.^[1]^ More specifically, our results show that overall satisfaction with doctors is more likely to influence PQHC among patients with lower levels of life satisfaction than among those with higher levels of life satisfaction.

Our results also show that privacy and perceived waiting time to be called back by the doctor after an examination and/or test are more likely to influence the PQHC among patients with lower levels of life satisfaction and happiness than among those with higher levels of life satisfaction and happiness.

However, no association has been found between patient satisfaction with emergency care and patient satisfaction with life in general.^[29]^ Research has also shown that high levels of life satisfaction may lead to higher expectations.^[29]^ One study showed that the type of treatment used affects patients’ levels of life satisfaction.^[30]^ Other studies have shown that the treatment approach and any resulting negative complications can negatively affect patients’ perceptions of their life satisfaction levels.^[31]^ A patient’s contribution to achieving their recovery goals is likely to be greater when their satisfaction levels are higher.^[32]^

In another study, Macrodimitris and Endler (2001) pointed out that patients diagnosed with chronic diseases believe that their health is more influenced by family members, friends, and health professionals than by chance or even themselves.^[33]^ While depressive symptoms may be a risk factor for decreased levels of life satisfaction, perceived social support from others may be a protective factor for high levels of perceived life satisfaction.^[34]^

Therefore, many researchers have highlighted the importance of medical and healthcare interventions that address the bidirectional relationship between emotional and physical well-being.^[35]^ In this sense, healthcare providers should integrate their medical care with early psychosocial interventions during follow-up care and at admission.^[34]^ It is also necessary to identify any psychological disturbances at an early stage, which can, in turn, aid in the implementation of effective treatment plans.^[34]^

However, regardless of all of these desirable measures of intervention, our results show that it is first important to identify an outcome variable and a certain predictor. This is because, in some cases, the level of happiness as a moderator is more important (contributes more) than the level of life satisfaction (e.g., in models with privacy and perceived waiting time to be called back by the doctor after the examinations and/or tests). Hence, in the moderation model with doctors, patients’ levels of happiness are totally unimportant, and the entire contribution is made by only the level of life satisfaction. Thus, these psychological notions can play different roles in models with different predictors.

## 5. Limitations

The data collection had some limitations as it was confined to 1 ED in 1 country. In addition, we only considered the Portuguese-speaking population who could answer the questions. We chose a sample distribution with a 5% margin of error rather than a lower margin of error due to time and financial constraints.

## 6. Conclusion

The main moderators between predictors and PQHC are 2 components of an individual’s psychological state (level of life satisfaction and level of happiness) and 1 specific patient characteristic (frequency of ED experiences). Separately, the contribution of these moderators in the models is quite small (the greatest contribution is made by the level of happiness, 3.0%, with privacy as a predictor). However, in light of the entire models with interaction effects, we found that the most significant effect belongs to the model with overall satisfaction with doctors as a predictor and the level of life satisfaction as a moderator, which explains 56.0% of the PQHC variation.

## Author contributions

**Conceptualization:** A.A.

**Data curation:** A.A.

**Formal analysis:** A.A. and S.M.

**Investigation:** A.A.

**Methodology:** A.A. and P.A.d.S.

**Project administration:** A.A.

**Resources:** A.A.

**Supervision:** A.A. and P.A.d.S.

**Validation:** A.A.

**Visualization:** A.A.

**Writing—original draft:** A.A.

**Writing—review and editing:** A.A., P.A.d.S., and S.M.
